# Dextromethorphan Attenuates Sensorineural Hearing Loss in an Animal Model and Population-Based Cohort Study

**DOI:** 10.3390/ijerph17176336

**Published:** 2020-08-31

**Authors:** Hsin-Chien Chen, Chih-Hung Wang, Wu-Chien Chien, Chi-Hsiang Chung, Cheng-Ping Shih, Yi-Chun Lin, I-Hsun Li, Yuan-Yung Lin, Chao-Yin Kuo

**Affiliations:** 1 Department of Otolaryngology-Head and Neck Surgery, Tri-Service General Hospital, National Defense Medical Center, Taipei 114, Taiwan; chw@ms3.hinet.net (C.-H.W.); zhengping_shi@yahoo.com.tw (C.-P.S.); lyc_1023@yahoo.com.tw (Y.-C.L.); yking1109@gmail.com (Y.-Y.L.); chefsketchup@hotmail.com (C.-Y.K.); 2 Graduate Institute of Medical Sciences, National Defense Medical Center, Taipei 114, Taiwan; 3 School of Public Health, National Defense Medical Center, Taipei 114, Taiwan; chienwu@mail.ndmctsgh.edu.tw (W.-C.C.); g694810042@gmail.com (C.-H.C.); 4 Department of Medical Research, Tri-Service General Hospital, National Defense Medical Center, Taipei 114, Taiwan; 5 Department of Pharmacy Practice, Tri-Service General Hospital, National Defense Medical Center, Taipei 114, Taiwan; lhs01077@gmail.com; 6 School of Pharmacy, National Defense Medical Center, Taipei 114, Taiwan

**Keywords:** dextromethorphan, noise, hearing loss, cochlea, synapse

## Abstract

The effect of dextromethorphan (DXM) use in sensorineural hearing loss (SNHL) has not been fully examined. We conducted an animal model and nationwide retrospective matched-cohort study to explore the association between DXM use and SNHL. Eight-week-old CBA/CaJ hearing loss was induced by a white noise 118 dB sound pressure level for 3 h. DXM (30 mg/kg) was administered intraperitoneally for 5 days and boost once round window DXM socking. In population-based study, we examined the medical records over 40 years old in Taiwan’s National Health Insurance Research Database between 2000 and 2015 to establish retrospective matched-cohort to explore the correlation between DXM use and SNHL. Using click auditory brainstem response (ABR), hearing threshold was measured as 48.6 ± 2.9 dB in control mice compared with 42.6 ± 7.0 dB in DXM mice, which differed significantly (*p* = 0.002) on day 60 after noise exposure with a larger ABR wave I amplitude in DXM mice. In human study, we used a Cox regression hazard model to indicate that a significantly lower percentage individuals developed SNHL compared with and without DXM use (0.44%, 175/39,895 vs. 1.05%, 1675/159,580, *p* < 0.001). After adjustment for age and other variables [adjusted hazard ratio: 0.725 (95% confidence interval: 0.624–0.803, *p* < 0.001)], this study also demonstrated that DXM use appeared to reduce the risk of developing SNHL. This animal study demonstrated that DXM significantly attenuated noise-induced hearing loss. In human study, DXM use may have a protective effect against SNHL.

## 1. Introduction

Hearing loss is a growing and alarmingly high burden in the world reporting from the Global Burden of Disease Studies and the third leading cause of years lived with disability [1,2]. Sensorineural hearing loss (SNHL) is primarily due to the degeneration of hair cells and spiral ganglion neurons in the cochlea resulting from acute and/or chronic events of extrinsic (e.g., ototoxic drugs, noise) and intrinsic causes (e.g., aging, genetic factors, congenital risk factors) [3,4]. The common causes of genetic, age-related, noise-induced, and drug-induced hearing loss display intriguing similarities in terms of particular cellular responses of the cochlear sensory cells comprising potential involvement of impaired mitochondrial function, ischemia, oxidative stress with reactive oxygen species, inflammation, apoptosis, autophagy, and/or necrosis [3].

Recently, a recent mechanism of SNHL is reported to be related with cochlear or auditory synaptopathy, which is caused by damage or loss to the synapses between inner hair cell (IHC) and spiral ganglion neuron (SGN) and causing deafferentation [5,6,7,8]. Cochlear synpatopathy is also involved in genetic, noise, ototoxicity and age-related hearing loss [7,8,9,10]. This mechanism may contribute to glutamate excitotoxicity involving *N*-methyl-d-aspartate (NMDA) receptor activation and related auditory nerve excitation [9,11,12]. NMDA receptor inhibition has been proposed as a pharmacologic approach for the treatment of synaptic hearing loss [13,14,15].

NMDA antagonists include ketamine, esketamine, dextromethorphan (DXM), phencyclidine, and dizocilpine [16,17]. DXM, an uncompetitive and low-affinity NMDA receptor antagonist, has been widely used as a nonopioid, nonnarcotic, and over-the-counter antitussive for over 50 years [16]. DXM has demonstrated considerable neuroprotective properties in numerous in vitro and in vivo models of central nervous system injury and neurodegenerative diseases [16,18]. DXM may exert its neuroprotective effects through multiple actions to inhibit glutamate neurotoxicity, inflammatory pathways, oxidative damage, calcium imbalances, and apoptosis with extremely similarity of pathophysiologies in SNHL and may play a role in sudden SNHL [3,16,19].

DXM has been reported to reduce neuronal damage or degeneration, cortical infarct volume and improve neurological functions in numerous animal models of stroke and traumatic brain injury (TBI) [18,20]. In 2010, the U.S. Food & Drug Administration approved the use of DXM in combination with quinidine for the treatment of pseudobulbar affect characterized by sudden and involuntary episodes of crying, laughing, or other emotional displays secondary to a neurological disease or brain injury, such as amyotrophic lateral sclerosis, stroke, Alzheimer’s disease, multiple sclerosis, Parkinson’s disease, and TBI [16]. To date, except our previous published paper, there is sparse study to explore the association between DXM use and preventing SNHL in inner ear of mouse model and human study [21]. Herein, we explore whether DXM, an NMDA antagonist, exhibits any potential effect against SNHL in both animal models and population-based study.

## 2. Materials and Methods

### 2.1. Animals and Noise Exposure

**Figure 1:**
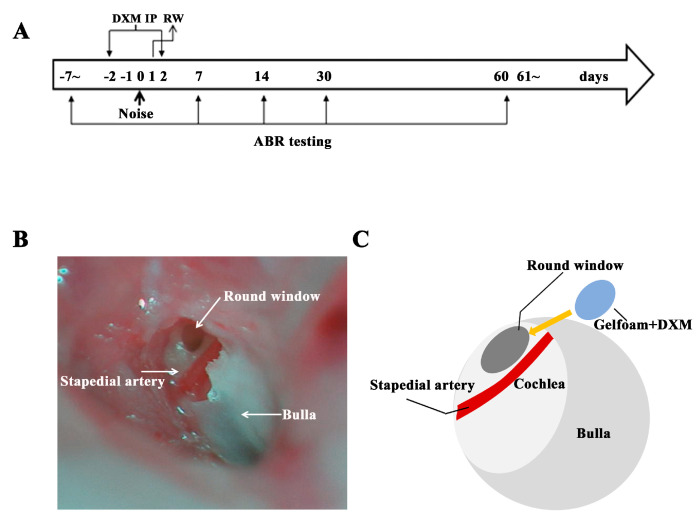
(**A**) Experimental schedule. Mice were exposed to noise on day 0. The auditory brainstem response (ABR) test was performed on days −7, 7, 14, 30, and 60. Dextromethorphan (DXM) was injected intraperitoneally (IP) on days −2 to 2 and boosted on day 1 through the round window approach. (**B**,**C**) Surgical approach for DXM round-window application.

All experiments were approved by the Institutional Animal Care and Use Committee of the National Defense Medical Center (Taipei, Taiwan, approval number IACUC-16-082). The animal care complied with the institutional guidelines and regulations. The schedule of the experiment is illustrated in Figure 1A. Male eight-week old CBA/CaJ mice are randomized into control and DXM group. In total, 22 mice were operated in the DXM group and 18 in the control group. The mice were anesthetized, placed in a soundproof booth with a loudspeaker (V12 HP, Tannoy, United Kingdom) mounted above the center of the cage, and both ears were exposed to white noise at a sound pressure level (SPL) of 118 dB for 3 h. The sound intensity inside the chamber was tested using a sound level meter to ensure minimal deviations of sound intensity.

### 2.2. DXM Application

DXM hydrobromide was purchased from Sigma-Aldrich (St. Louis, MO, USA). Each dose (30 mg/kg) was injected intraperitoneally once/per day for 5 contiguous days starting 2 days before noise exposure. The next day after noise exposure, the animals’ unilateral round windows were surgically exposed to boost DXM soaking to enhance the possible therapeutic effect and future application of therapy clinically by intratympanic injection. The control group was administered phosphate-buffered saline (PBS) during the same surgical procedure (sham). The animals were anesthetized with ketamine (100 mg/kg, intraperitoneally) and xylazine (10 mg/kg, intraperitoneally). Half the initial dose of anesthesia was administered on the reappearance of blinking or the withdrawal reflex in the animals. A posteroinferior skin incision was made in the retroauricular area behind the right ear. The underlying muscles and facial nerve were separated by blunt dissection to expose the middle compartment of the bulla, and the round window niche was exposed through a small opening. A small piece of gelfoam soaked with DXM was placed into the round window and bulla (Figure 1B,C). The gelfoam about 1 mm in size soaked with 5 ul DXM (3.75 mg/mL) was administered to the round window and solved within 2 weeks. The incision was closed with nonabsorbable sutures, and the animals were transferred onto a homeothermic blanket at 39.8 °C for the recovery period. 

### 2.3. Auditory Brainstem Response Recording

The animals’ auditory function in the surgical ear was assessed by recording the auditory brainstem responses (ABRs) as previously described [22]. In brief, the mice were anesthetized and kept warm with a heating pad in a sound-attenuating chamber. Subdermal needle electrodes were inserted at the vertex (positive), below the pinna of the ear (negative), and at the back (ground) of the mice. Specific stimuli (clicks and 8-, 12-, 16-, 20-, 24-, 28-, and 32-kHz tone bursts) were generated using the SigGen software program (Tucker-Davis Technologies, Gainesville, FL, USA) and delivered to the external auditory canal. The average responses to 1024 stimuli at each frequency were obtained by reducing the sound intensity in 5-dB steps until the threshold was reached. The resulting ABR thresholds were defined as the lowest intensity at which a reproducible deflection in the evoked response trace could be recognized. The ABR wave I peak-to-peak amplitude was computed through an offline analysis of the stored waveforms.

### 2.4. Population-Base Database

This study employed a retrospective matched-cohort design. We acquired medical records over 40 years old from the National Health Insurance Research Database (NHIRD, an outpatient and hospitalization longitudinal health insurance database in Taiwan) between 1 January 2000 and 31 December 2015, to establish matched cohorts with and without DXM use, using a propensity score method at a ratio of 1:4 by sex, age, and index year. Patients were diagnosed with sensorineural hearing loss (SNHL) according to ICD-9-CM code (389.1x). We excluded all patients with a prior hearing loss diagnosis and those who had been prescribed DXM before 2000. 

Gender, age, covariates, and comorbidities were assessed and analyzed in this study. We defined catastrophic illness using the definition of the Ministry of Health and Welfare in Taiwan, which contains 30 categories that the patients can apply for a certificate to become exempt from copayments for healthcare costs related to catastrophic illness [23].

Data used in this study are managed and stored by the Health and Welfare Data Science Center. Researchers can obtain the data through formal application to the Health and Welfare Data Science Center, Department of Statistics, Ministry of Health and Welfare, Taiwan (http://dep.mohw.gov.tw/DOS/np-2497-113.html).

The Institutional Review Board of Tri-Service General Hospital approved this study (TSGHIRB no. 2-105-05-082) and waived the requirement of written informed consent to access the NHIRD.

### 2.5. Duration of DXM Use and Sensitivity Analysis

The DXM use in this population-based study was prescribed as systemic application. The data of the defined daily dose (DDD) were obtained from the WHO Collaborating Centre for Drug Statistics Methodology (https://www.whocc.no/), and the duration of the usage of DXM was calculated by dividing the cumulative doses by the DDD of DXM. Duration of DXM use was categorized into three subgroups of ≤30, 30~90 and >90 days. The sensitivity test for duration of DXM use was analyzed for risk of developing SNHL using Cox regression model. 

### 2.6. Statistical Analysis

For animal study, the statistical analysis was performed through one-way analysis of variance between control and DXM groups. The results are expressed as the mean ± standard error of the mean. The differences were considered significant at *p* < 0.05.

For human study, all data analyses were performed using IBM SPSS for Windows, version 22.0 (IBM Corp., Armonk, NY, USA). The Chi-square test and Fisher’s exact test were used to compare the difference of categorical variables, and Student’s t test was used to compare the difference of continuous variables between with DXM use and without DXM use. Multivariate Cox proportional hazards regression was used to determine the risk of SNHL, and the results are presented as a hazard ratio (HR) with 95% confidence interval (CI). The difference in risk of SNHL for patients with or without DXM use was estimated using the Kaplan–Meier method with a log-rank test. A two-tailed *p* value < 0.05 was considered statistically significant.

## 3. Results

### 3.1. Animal Study

**Figure 2:**
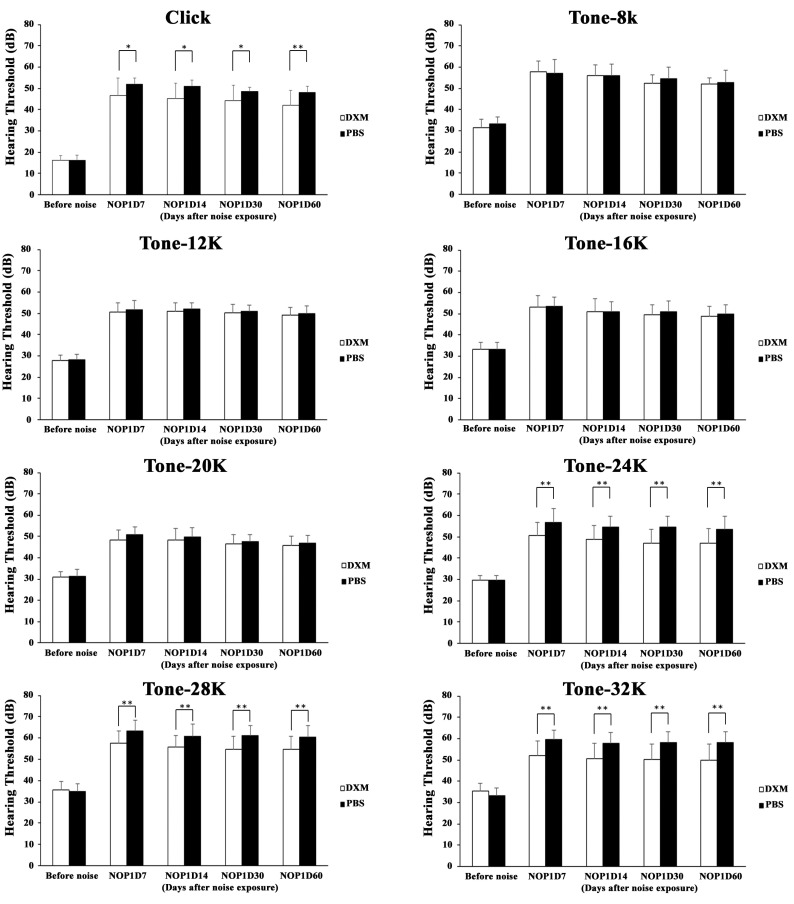
Click and tone burst ABR for hearing evaluation at various time points. The hearing threshold obtained through click ABR (42.6 ± 7.0 dB, *n* = 22) in the DXM group was significantly (*p* = 0.002) better than that (48.6 ± 2.9 dB, *n* = 18) in the control group after noise exposure. In terms of tone burst ABR, the hearing threshold between 24 and 32 kHz in the DXM group was significantly better than that in the control group after noise exposure. * *p* < 0.05, ** *p* < 0.01.

A permanent threshold shift was produced after exposure at an SPL of 118 dB for 3 h. A 30 mg/kg dose of DXM was injected intraperitoneally from 2 days before noise exposure to 2 days after noise exposure. We noted that surgery to access the round window did not affect the hearing threshold in mice which was compatible with previous literature [24]. Subsequently, we boosted DXM soaking in the round window the next day after noise exposure. The hearing threshold was measured using the ABRs on day 7, 14, 30, and 60 after noise exposure (Figure 2). From 7 days after noise exposure, the hearing threshold differed significantly between both groups. The hearing threshold was measured as 48.6 ± 2.9 dB through the click ABR in the control group (*n* = 18) compared with 42.6 ± 7.0 dB in the DXM group (*n* = 22) on day 60 after noise exposure. The tone burst ABR at frequencies of 24, 28, and 32 K had a significantly lower hearing threshold in the DXM group than in the control group. Although the mice were anesthetized with another NMDA antagonist (ketamine) that may have affected the experimental effect, the difference can be neglected because the same procedure was used in both groups.

**Figure 3:**
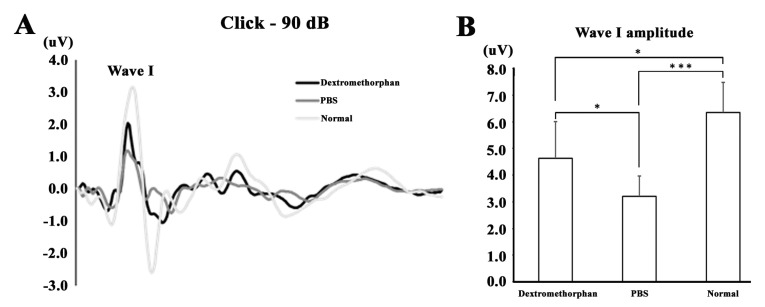
(**A**) ABR waveforms appeared in normal mice without noise exposure, and in the DXM and PBS mice groups with noise exposure. (**B**) ABR wave I amplitude was higher in the DXM group compared with the PBS group. * *p* < 0.05, *** *p* < 0.001.

In recent studies, the synapse between inner hair cells and spiral ganglion neurons has been reported to mediate hearing transduction [25]. The amplitude of ABR wave I can be represented as the number of synaptic ribbons [6]. We also analyzed the raw ABR data from the mice on day 60 for wave I amplitude. The data obtained through the click ABR indicated that the morphology of waveforms were better and wave I amplitude was higher in the DXM group compared with the PBS group (Figure 3). This result may reflect the potential effect of DXM treatment with preserved synaptic complexes.

### 3.2. Population-Based Human Study

**Figure 4:**
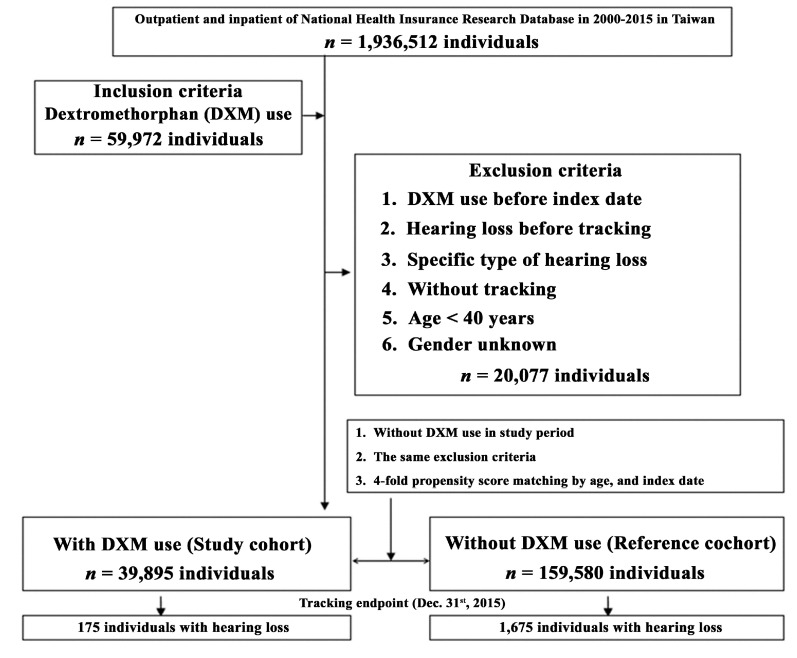
Flowchart of study sample selection.

Based on the data used in this study from 1 January 2000 to 31 December 2015, 39,895 individuals with DXM use were included and a matched 159,580 individuals without DXM use were selected as the control group (Figure 4). At the end of the follow-up period (Table 1), 175 individuals with DXM use (0.44%, 175/39,895) and 1675 without DXM use (1.05%, 1675/159,580) had developed SNHL, indicating a significantly lower incidence of hearing loss among those with DXM use (*p* < 0.001). The average follow-up period was 9.90 ± 9.28 years, and the average period for developing hearing loss was 4.87 ± 5.54 years. Significantly lower percentages of ischemia heart disease (IHD) (12.94% vs. 13.70%; *p* = 0.021) and depression (0.48% vs. 0.65%; *p* = 0.026) were found in individuals with DXM use, compared with the control group.

**Figure 5:**
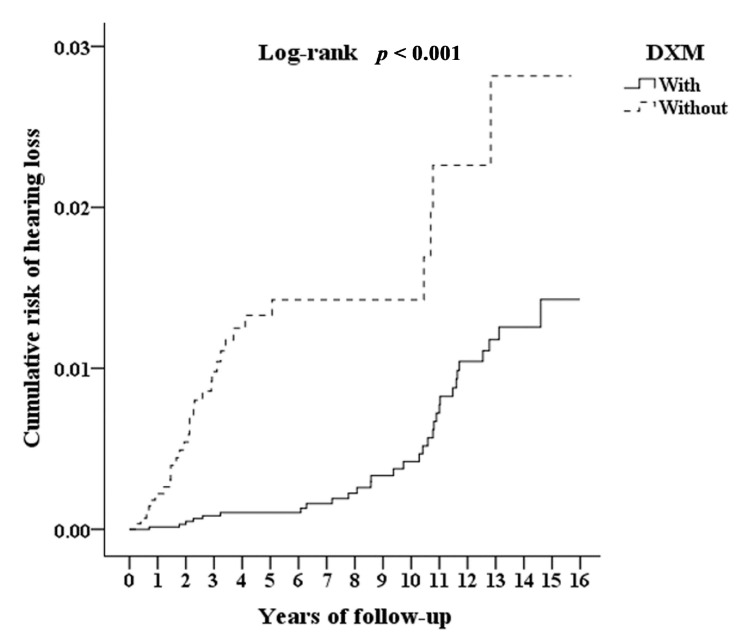
Kaplan–Meier for cumulative risk of hearing loss among individuals aged > 40 with or without DXM use.

The cumulative incidence curve of SNHL for the cohort with DXM use was significantly lower than for the control cohort, following adjustment for age and other variables (Figure 5; log-rank test, *p* < 0.001).

After adjustment for age, sex, and comorbidities in the Cox proportional hazard regression, DXM use was still significantly associated with a decreased risk of hearing loss, with an adjusted HR of 0.725 (95% CI, 0.624–0.803; *p* < 0.001; Supplementary Table S1). Patients with catastrophic illness, older patients, diabetes mellitus, hypertension, stroke, chronic kidney disease, autoimmune diseases, IHD, pneumonia, head injury, and chronic liver disease had a significantly higher risk of SNHL.

We investigated the sensitivity test for duration of DXM use and the test demonstrated a lower risk of developing SNHL was associated with longer duration of DXM use (Table 2).

## 4. Discussion

Our data demonstrated and explored that DXM, an NMDA antagonist, had the ability to attenuate noise-induced hearing loss (NIHL) by improving the hearing threshold and wave I amplitude, as indicated through ABR testing, and also potentially decrease the risk of SNHL after adjusting by Cox regression analysis in a 16-year follow-up nationwide population-based study. To the best of our knowledge, this is the first report to explore the preventive effect of DXM use in hearing loss in both animal and human study.

Some NMDA antagonists have been used to investigate the protective effect in the inner ear using animal models of noise [11,12,14], ischemia [17,26,27], and ototoxicity [10,12,28]. Jäger et al. demonstrated that dizocilpine maleate (MK-801) had a protective effect against NIHL, but the effect was limited to a specific frequency [11]. Duan et al. also presented MK-801 as a means to substantially limit both NIHL and swelling of dendrites under inner hair cells [12]. Bing et al. reported that esketamine hydrochloride gel (AM-101) may reduce the noise-induced loss of synaptic ribbons, but it had no protective effect against NIHL [14]. Tabuchi et al. reported that ketamine and dextromethorphan, but not MK-801 have protective effect on cochlear ischemia dysfunction [17]. MK-801 and ifenprodil resulted in the protection of aminoglycoside-induced ototoxicity [12,28]. There was still sparse study to explore the effect of DXM use in common hearing loss animal models.

Given that NMDA receptor antagonists tend to have negative central nervous system side effects, such as hallucinations or anesthesia, their clinical potential has been limited [14]. In the present study, we demonstrated that DXM, widely used and available in numerous over-the-counter cough and cold preparations worldwide [16], also has a protective effect against NIHL. The result was compatible to our previous report in a NIHL rat model using 4-[^18^F]-ADAM/micro-PET and showed DXM prevent hearing loss and preserved brain serotonin transporter function [21]. This effect also has been proven previously that DXM moderately ameliorated the compound action potential threshold shift in cochlear dysfunction induced by transient ischemia [17].

Based on aforementioned results, DXM has the potential to be a promising therapeutic intervention, we directly explored the association between DXM use and the risk of SNHL using Taiwan’s NHIRD database. After adjusting variables by multiple Cox regression analysis, the use of DXM significantly reduced the risk of SNHL compared with matched-cohort control group. DXM use is frequently prescribed for patients when they might catch cold in Taiwan. We used cumulative DDD (cDDD) of DXM to analyze the association between DXM use and risk of SNHL. The result revealed longer duration of DXM use (larger amount of cDDD) had more significantly decreased the risk of SNHL. Herein, we further provided the evidence to support the finding that the use of DXM has a strong impact in preventing SNHL.

The mechanism for the preventive effect of DXM from hearing loss may be through the inhibition of glutamate-induced excitotoxicity at the highly active synapse due to NMDA receptor activation, dendritic swelling, and the production of reactive oxygen species [9,29]. Glutamate-induced excitotoxicity was recently thought as an instigating factor of cochlear synaptopathy [8,9]. The use of DXM can have the capacity to protect synaptic damage in acute noise-exposed hearing loss.

In recent years, a hidden hearing loss theory has advocated that synaptic loss is the primary pathology even with only temporary threshold shifts, and that this synaptic loss is independent from both IHC and SGN loss [6,30]. Cochlear synaptopathy may contribute to hearing impairment in millions of people [8,31]. If these damaged synaptic connections can be maintained or restored by any potential drugs or other therapeutic management, it could undoubtedly improve the hearing function [32]. Some reports have evidenced that neurotrophins, such as neurotrophin-3 and brain-derived neurotrophic factors, can offer protective effects against noise trauma in animals [24,33,34]. DXM would be an effective and alternative drug to apply in further human studies on NIHL (e.g., recreational or military gun shooting) or age-related SNHL.

Our study had some limitations. In animals, it was not clear which approach (intraperitoneal injection or round window soaking) resulted in the therapeutic effect because we combined both approaches and we did not assess the immunohistochemistry staining for synaptic ribbon. In humans, we were unable to reduce non DXM contributing factors in the retrospective-matched cohort study. This is because: (1) we cannot assess a patient’s history and cause of SNHL according to the diagnosis by using the ICD-9-CM code (389.1x), (2) the severity of hearing threshold of individuals as determined by audiometry was not available in the current NHIRD database, and (3) the timing of DXM application was inaccessible as it was not prescribed for treatment of SNHL. All of these factors may have contributed to the otoprotective effect of DXM in this study. Future clinical trials would have to sufficiently reduce non DXM contributing factors to confirm our findings.

## 5. Conclusions

Our study demonstrated that DXM significantly attenuated noise-induced hearing loss and may have a protective effect against SNHL. The effect may be through synaptic regulation. Therefore, to develop a novel effect of pharmaceutical medicine to prevent cochlear synaptopathy is practical to the hearing preservation program. DXM can be an alternative medication applying in future clinical trials for SNHL.

## Figures and Tables

**Table 1:** Characteristics of study in the endpoint during the follow-up period from 1 January 2000 to 31 December 2015.

DXM Use	With		Without		*p*
	*n*	%	*n*	%	
Total	39,895	20.00	159,580	80.00	
Hearing loss	175	0.44	1675	1.05	<0.001
Variables					
Gender					0.999
Male	20,121	50.43	80,484	50.43	
Female	19,774	49.57	79,096	49.57	
Age (years)	55.28 ± 26.45		54.70 ± 25.51		<0.001
Catastrophic illness	2671	6.70	12,104	7.58	<0.001
DM	3884	9.74	16,011	10.03	0.076
HTN	9112	22.84	35,142	22.02	<0.001
Depression	1097	2.75	4048	2.54	0.016
Insomnia	1845	4.62	6822	4.27	0.002
Stroke	2111	5.29	8705	5.45	0.197
CKD	4344	10.89	17,035	10.67	0.217
Hyperlipidaemia	1184	2.97	4499	2.82	0.111
Epilepsy	429	1.08	1375	0.86	<0.001
AID	3245	8.13	13,401	8.40	0.088
IHD	2675	6.71	12,604	7.90	<0.001
COPD	2973	7.45	12,801	8.02	<0.001
Pneumonia	5671	14.21	20,754	13.01	<0.001
Head injury	6881	17.25	26,279	16.47	<0.001
Asthma	4213	10.56	17,987	11.27	<0.001
Alcohol abuse/dependence	381	0.96	1275	0.80	0.002
Tobacco abuse/dependence	291	0.73	1248	0.78	0.282
CLD	4041	10.13	16,124	10.10	<0.001
Parkinson’s disease	900	2.26	3251	2.04	0.006
Urbanization level					<0.001
1 (The highest)	12,121	30.38	44,026	27.10	
2	13,986	35.06	54,521	33.57	
3	5111	12.81	20,782	12.79	
4 (The lowest)	8677	21.75	43,101	26.54	
Level of care					<0.001
Hospital center	14,562	36.50	46,128	28.91	
Regional hospital	15,756	39.49	65,117	40.81	
Local hospital	9577	24.01	48,335	30.29	

*p*: Chi-square/Fisher exact test on category variables and *t*-test on continue variables; DM = diabetes mellitus; HTN = hypertension; CKD = chronic kidney disease; AID = autoimmune diseases; IHD = ischemia heart disease; COPD = chronic obstruction pulmonary disease; CLD = chronic liver disease.

**Table 2:** Factors of hearing loss among different duration of DXM by using Cox regression.

DXM Dose	Population	Events	PYs	Rate (per 10^5^ PYs)	Adjusted HR	95% CI	95% CI	*p*
Without	159,580	1675	2,012,465.11	83.23	Reference			
With	39,895	175	513,401.02	34.09	0.725	0.624	0.803	<0.001
1–30 days	8423	43	114,267.35	37.63	0.797	0.684	0.888	<0.001
31–90 days	19,450	88	248,640.12	35.39	0.756	0.642	0.834	<0.001
≥91 days	12,022	44	150,493.55	29.24	0.622	0.531	0.697	<0.001

PYs = Person-years; Adjusted HR = Adjusted Hazard ratio: Adjusted for the variables listed in Table 1; CI = confidence interval; DXM = dextromethorphan.

## Supplementary Materials

The following are available online at https://www.mdpi.com/1660-4601/17/17/6336/s1, Table S1: Factors of hearing loss by using Cox regression.
