# Supplementary material for: Dextromethorphan Attenuates Sensorineural Hearing Loss in an Animal Model and Population-Based Cohort Study
Source: Int J Environ Res Public Health. 2020 Aug 31;17(17):6336. doi: 10.3390/ijerph17176336 (PMC7504445; doi:10.3390/ijerph17176336)
Supplement: Supplementary file 1 [file ijerph-17-06336-s001.pdf]

Supplementary Table S1. Factors of hearing loss by using Cox regression

Variables (with/without)	Adjusted HR	95% CI	95% CI	*P*
**DXM use**	0.725	0.624	0.803	<0.001
**Gender (Male/Female)**	1.264	0.896	1.594	0.298
**Age (yrs)**	1.801	1.277	2.980	<0.001
**Catastrophic illness**	1.989	1.556	2.870	<0.001
**DM**	2.659	1.896	3.454	<0.001
**HTN**	1.852	1.113	2.454	<0.001
**Depression**	1.254	0.897	1.488	0.074
**Insomnia**	1.246	0.456	2.706	0.298
**Stroke**	2.105	1.124	2.886	<0.001
**CKD**	2.785	1.865	3.798	<0.001
**Hyperlipidaemia**	1.402	0.795	1.986	0.270
**Epilepsy**	0.986	0.412	1.596	0.712
**AID**	2.121	1.425	2.986	<0.001
**IHD**	1.597	1.124	1.986	<0.001
**COPD**	0.896	0.442	1.886	0.245
**Pneumonia**	1.896	1.111	2.565	<0.001
**Head injury**	3.154	1.298	5.010	<0.001
**Asthma**	0.896	0.225	1.898	0.344
**Alcohol abuse / dependence**	1.382	0.752	1.866	0.288
**Tobacco abuse / dependence**	1.124	0.652	1.782	0.372
**CLD**	1.896	1.562	2.453	<0.001
**Parkinson's disease**	1.265	0.796	1.588	0.521
**Level of care**				
Hospital center	1.782	1.335	1.972	<0.001
Regional hospital	1.486	1.254	1.653	<0.001
Local hospital	Reference			

Adjusted HR = Adjusted hazard ratio: Adjusted variables listed in the Table 1;
CI = confidence interval
